# Detection of Specific Volatile Organic Compounds in *Tribolium castaneum* (Herbst) by Solid-Phase Microextraction and Gas Chromatography-Mass Spectrometry

**DOI:** 10.3390/foods12132484

**Published:** 2023-06-25

**Authors:** Shaoyun Han, Ke He, Jing An, Mengmeng Qiao, Runhui Ke, Xiao Wang, Yang Xu, Xiuying Tang

**Affiliations:** 1 College of Engineering, China Agricultural University, Beijing 100083, China; hanshaoyun2022@163.com (S.H.);; 2 Sinolight Inspection& Certification Co., Ltd., Beijing 100083, China

**Keywords:** *Tribolium castaneum*, volatile organic compounds, solid-phase microextraction (SPME), gas chromatography-mass spectrometry (GC-MS)

## Abstract

The red flour beetle, *Tribolium castaneum* (Herbst) (Coleoptera: Tenebrionidae), is a major storage pest that could lead to a wide range of damage. Its secretions have a significant impact on the quality of stored grain and food, leading to serious food safety problems such as grain spoilage and food carcinogenesis. This study investigates new detection techniques for grain storage pests to improve grain insect detection in China. The primary volatile organic chemicals (VOCs) in these secretions are identified using headspace solid-phase microextraction (HS-SPME) coupled with gas chromatography-mass spectrometry (GC-MS). The specific VOCs that are unique to *T. castaneum* are selected as criteria for determining the presence of *T. castaneum* in the granary. To obtain more specific VOCs, experiments were designed for the analysis of *T. castaneum* samples under different extraction times, two types of SPME fibers and two GC-MS devices of different manufacturers. The experimental results showed that 12 VOCs were detected at relatively high levels, seven of which were common and which were not detected in other grains and grain insects. The seven compounds are 1-pentadecene, 2-methyl-p-benzoquinone, 2-ethyl-p-benzoquinone, 1-hexadecene, cis-9-tetradecen-1-ol, m-cresol and paeonol. These seven compounds can be used as volatile markers to identify the presence of *T. castaneum*, which could serve as a research foundation for the creation of new techniques for *T. castaneum* monitoring.

## 1. Introduction

The red flour beetle, *Tribolium castaneum* (Herbst) (Coleoptera: Tenebrionidae), is one of the most common pests affecting flour and flour products. It also widely exists in stored cereal grains, nuts, Chinese traditional material crops, oil, distiller’s yeast and other durable agricultural products all over the world [1,2,3]. The ability of seeds, if eaten by *T. castaneum*, to germinate is undermined. Various secretions produced by the pest can also cause serious contamination effects on the product infested, causing the damaged grain to agglomerate, develop mildew, and smell. Therefore, the presence of these pests can affect the quality and nutritional standards of food and may pose a serious threat to human health [1,2,3,4]. Even the presence of a single pest during the storage of flour or flour products cannot be ignored to cause harm [5]. Therefore, the frequent sampling testing has become a necessary means to detect the existence of pests in stored products. It is important to control grain pests by using new and effective pesticides [6,7,8,9], but identifying the extent to which stored grain is infected by pests is the basis for control. The sooner you find out, the fewer pesticides you might use and the fewer grain storage losses it will incur. Therefore, it is necessary to detect stored grain pests quickly. The traditional grain pest detection methods are mainly manual, but they cannot meet the demand for rapid detection of grain storage pests due to tedious and time-consuming operations. As a result, exploring a new way of rapid, efficient and accurate detection of grain storage pests has become a research focus for many scholars. Currently, the main new grain pest detection methods are near-infrared spectroscopy [10,11], the image recognition method [12,13], electronic nose (E-nose) [14,15,16], soft x-ray imaging [17,18], etc. The image recognition method cannot identify pests inside the grain; the near-infrared spectrum is sensitive to the moisture content of the grain; and the x-ray imaging is still a challenge for soft food. E-nose detection has the advantages of being fast, able to perform in real-time, low cost, easy to operate and portable, but the selection of odor sensors in E-nose devices is difficult because there are no sensors on the market that specifically differentiate grain pest odor. Therefore, it is of great significance to find the main volatile compounds unique to grain pests for the manufacture, optimization and improvement of the detection accuracy of the E-nose.

Recently, some researchers have been looking for the unique VOCs of grain pests in an attempt to detect grain pests [19,20,21]. HS-SPME in conjunction with GC-MS has been widely employed to detect VOCs from stored grain insects in cereals [22,23,24,25]. Among them, Seitz and Sauer explored the changes in volatile compounds in healthy sorghum and sorghum infested with a variety of grain storage pests by using GC-MS [26]. Seitz and Ram identified that the metabolites of *Rhyzopertha dominica* (Fabricius) in wheat comprise a large number of volatile alcohols and esters [27]. Arnaud found that 4,8-dimethyldecanal (DMD) was a common aggregation pheromone of three flour pests [28].

Researchers have found a wide spectrum of VOCs from grains, pests and microorganisms in some stored grains; these volatile chemicals comprise a wide range, including alcohols, ketones, aldehydes, esters, carboxylic acids, compounds of sulfur and nitrogen, and macromolecular esters and terpenes. Related studies have shown that VOCs of *T. castaneum* are the part of the secretions detected above, of which benzoquinone has been identified as one of the secretions. M.L. Villaverde concluded that three compounds, 2-methyl-p-benzoquinone, 2-Ethyl-p-benzoquinone and 1-pentadecene, accounted for more than 90% of the total volatile mixture of *T. castaneum* [22]. Nikola Đukić concluded that 1-pentadecene was present in wheat bran infested with *T. castaneum*, but was not found in uninfested bran [29]. Additionally, the low, medium and high concentrations of 1-pentadecene were attractive, neutral and repulsive to *T. castaneum*, respectively. In conclusion, most researchers agree that the main volatile compounds of *T. castaneum* are 1-pentadecene, 2-methyl-p-benzoquinone and 2-ethyl-p-benzoquinone. However, it is insufficient to use only three compounds as the specific VOCs of the grain pests. Therefore, it is crucial to explore and extract specific volatile compounds that can accurately characterize *T. castaneum* with HS-SPME/GC-MS.

The research has shown that the extraction of VOCs from *T. castaneum* using HS-SPME/GC-MS can be affected by the experimental environment and experimental conditions in which the sample is located. Environmental factors include insect food, nutritional variety, sex, age, number, wetness, temperature, and the fact that different species of insects living together also affect the production of volatile compounds in insects [30,31,32,33,34]. The setup of the above experimental environment can be strictly adjusted artificially according to the experimental requirements. Furthermore, the selection of important components of the GC-MS instrument in the experimental conditions and the data analysis process can affect the detection results, such as the selection of the solid-phase microextraction fiber membrane type, the extraction time of the sample volatile compounds, the resolution time, the temperature and the version of the mass spectrometry data matching library. Particularly, different SPME fiber types and extraction times have a direct impact on the kind and quantity of chemicals that reach the GC-MS instrument, and the detection results can change depending on the performance indicators of various instruments. Researchers have carried out pertinent studies to examine the optimal matching of experimental methodologies. Niu used a three-phase solid-phase microextraction fiber membrane (DVB/CAR/PDMS), an Agilent gas chromatograph with a non-polar Rxi^®^-5 ms and a polar Stabilwax^®^ capillary column, a NIST08 mass spectrometry library, and an extraction time of 4 h for the detection of VOCs in wheat for *Rhyzopertha dominica* (Fabricius) and in flour for *T. castaneum* [24,25]. Lu, SL used a Shimadzu gas chromatograph with a non-polar DB-5MS capillary column and NIST mass spectrometry library to study the odorous substances of wheat after different degrees of infection with *T. castaneum* [35]. M.L. Villaverde treated *T. castaneum* samples at three different temperatures with three SPME fibers (CAR/PDMS, PDMS, DVB/PDMS), a Hewlett-Packard gas chromatograph, a non-polar DB-5 capillary column, and a NIST98/EPA/NIH mass spectrometry library, with an extraction time of 15 min; it was concluded that two-phase SPME fiber (CAR/PDMS) had the highest overall sensitivity to VOCs from *T. castaneum* [22]. Furthermore, 2-methyl-p-benzoquinone, 2-ethyl-p-benzoquinone and 1-pentadecene were detected by all three SPME fibers. Nikola Đukić used an Agilent gas chromatograph with a non-polar HP-1 capillary column and NIST08 mass spectrometry library to study the effect of different concentrations of 1-pentadecene on the repellent or eliciting behavior of *T. castaneum* [29]. Differences in extraction time, SPME fibers, and GC-MS instrument manufacturers can cause different results, so it is critical to explore the polar chromatographic columns, the optimal extraction time, different SPME fibers, and the use of GC-MS instruments from different manufacturers for comparison to screen stable characteristic compounds.

To sum up, in this study, an HS-SPME/GC-MS technique was adopted to analyze and screen the main VOCs that could better characterize the information of *T. castaneum*. The research contents include three aspects: (1) the preparation of samples and selection of test components; (2) to investigate the effect of different extraction times on the qualitative analysis of the volatile compounds of *T. castaneum*; (3) the preference of two GC-MS instruments and two materials of SPME fibers based on polar columns for specific volatile markers of *T. castaneum*. The condition of grain storage pests can be indicated by the types and concentrations of volatile markers; thus, this study also provides a theoretical basis for improving the detection method of grain storage pests.

## 2. Materials and Methods

### 2.1. Insects

The laboratory population of *T. castaneum* used in the study were obtained from grain silos and cultured in the laboratory for several generations. Adults of *T. castaneum* were placed in 800 mL bottles which were sealed with netted lids. Newly harvested wheat (Jiangsu Province) was selected, washed and dried to 12.5 ± 0.5% moisture content, processed into whole flour by crusher, stored in a −4 °C refrigerator for 7 days, and then kept in a 4 °C freezer for storage. Whole wheat flour was used as the feed for *T. castaneum*. The entire rearing process was maintained at 28 ± 2 °C and 65 ± 5% relative humidity (RH) and in constant darkness. The age of the adults used in the experiments was 2–4 weeks. Insects were separated from whole flour using a sieve, and then active adults were selected for the experiment using a brush.

### 2.2. Volatile Organic Compounds’ Collection

The capacity of the headspace bottle is 50 mL. The lid is a polypropylene opening cap with a Teflon cover with a rubber septum. The materials of the sealing gasket are polytetrafluoroethylene (PTFE) and silicone. Headspace bottles are cleaned and autoclaved to ensure that there is no odor in the vial. Five adults of *T. castaneum* alone were placed in each vial (clean headspace bottle). Although one pest provided a detectable signal, as the initial analysis showed high individual variability, the pests were pooled for VOC measurements. All samples were sealed and stored at 28 °C for 24 h before testing and kept away from light. A variety of secretions were produced by *T. castaneum* (starving state) and some of these volatiles entered the sealed headspace bottle to form a mixture of gases. The VOCs secreted were sampled from the headspace (HS) of the vial by using a manual sampler, corresponding to the gas phase in contact with the pests. A manual sampler was used to collect VOCs in the air at the top of the vials. The manual SPME Holder and SPME Fiber Assembly purchased from Supelco (Bellefonte, PA, USA) were used in this study. 

### 2.3. SPME-GCMS Equipment and Methods

In this study, 50/30 μm Divinylbenzene/Carboxen/Polydimethylsiloxane (DVB/CAR/PDMS), 75 μm Carboxen/Polydimethylsiloxane (CAR/PDMS), and a polar column (DB-WAX) were used. An internal standard (cyclohexanone) was introduced to reduce errors when comparing data between different experiments for analysis. The identification of VOCs is based on chromatographic (retention time and Kovats index) and spectroscopic (mass spectrometry interpretation and database searches) criteria, as well as retention index and mass spectrum comparisons with real samples. The spectroscopic and chromatographic data were acquired on a gas chromatograph, GC 7890B (Agilent Technologies, Palo Alto, CA, USA), equipped with a mass selective detector MSD 5977B (electron impact ionization, EI, 70 eV; Agilent Technologies), and a split/splitless injector (1:50 split ratio). The MassHunter workstation was used for qualitative analysis and the relatively high levels of compounds were compared and identified individually using the NIST14 mass spectrometry library. Mass spectra and reconstructed chromatograms (TIC) were obtained by automatic scanning in the mass range *m/z* 35–400 u. A polar column (J&W DB-WAX) was used, which was 30 m × 0.25 mm × 0.25 μm in size. The injector was operated in the splitless mode at 250 °C. For the DB-WAX column, the oven temperature was programmed from 45 °C (5 min) to 220 °C (5 min) at 5 °C/min. The total operating time was 45 min. The temperatures of the ionization chamber and the transmission line were set at 230 and 285 °C, respectively. The carrier gas used was helium at a constant airflow of 1.0 mL/min [23,25,36].

The spectroscopic and chromatographic data were obtained with a gas chromatograph, GCMS-QP2020 NX (SHIMADZU, Kyoto, Japan) with LabSolutions Insight software, which included the spectral library NIST. The volatiles were identified by comparison of the mass spectrum with the NIST14 mass spectrometry library. A polar column (J&W DB-WAX) was used, which was 30 m × 0.25 mm × 0.25 μm in size. The injector was operated in the splitless mode at 250°C. For the DB-WAX column, the oven temperature was programmed from 45 °C (5 min) to 105 °C (5 min) at 5 °C/min. After that, the temperature was increased to 150 °C (1 min) again at 3 °C/min. After that, the temperature was increased to 230 °C (2 min) again at 6 °C/min. The total operating time was 48.3 min. The temperatures of the ionization chamber and the transmission line were set at 230 and 285 °C, respectively. The carrier gas used was helium at a constant airflow of 1.0 mL/min [23].

Preparation of internal standard solution: The chromatographically pure cyclohexanone standard (purity: 99.9%; density is 0.947 g/mL at 25 °C, Henan North Weiye Measurement Technology Co., Ltd., Xinxiang, China); Microliter Syringes (5 μL). The cyclohexanone standard (2 μL) was thoroughly mixed with deionized water (19.8 mL) and prepared as the 95.8 mg/L internal standard solution. An amount of 5 μL of the internal standard solution was added to the sample bottle using a micro-injector before each sample rested for 24 h. To avoid any accidental eating by insects, the internal standard solution was left to dangle on the glass vials inside wall.

### 2.4. Statistical Analysis 

The variations of VOC concentrations, the replicate samples and injections in comparison with average readings were analyzed by using Microsoft Excel 2019 and OriginPro 2023 (Learning Edition, OriginLab Corporation, Northampton, MA, USA). The signal values (TIC peak area) of each compound from three experiments were averaged as the signal value of this compound at that pest density.

Due to the individual differences of pests in the experimental samples and the error of the instrument itself, the response intensity of the peaks of various compounds’ peaks measured in different experiments also vary to some extent. To further reduce the above errors, the method of adding internal standards and optimizing the correction factors was used. The optimized correction factor formula is as follows:

(1) $$f_{i}=\frac{A_{i}}{\overset{-}{A}}\;\mathrm{or}\;\frac{h_{i}}{\overset{-}{h}},$$

$A_{i}$: Peak area of the internal standard in the *i*-th experiment

$\overset{-}{A}$: Average of peak areas of internal standards in all experiments

$h_{i}$: Peak height of the internal standard in the *i*-th experiment

$\overset{-}{h}$: Average of peak heights of internal standards in all experiments

The peak area of the compound in the *i*-th experiment × $f_{i}$. This formula’s purpose is to guarantee that the peak area of the internal standard is the same in every experiment and that the peak areas of the other compounds vary by a factor of $f_{i}$.

## 3. Results

### 3.1. Agilent GC-MS Analysis

Two sets of experiments, DVB/CAR/PDMS + Agilent GC-MS and CAR/PDMS + Shimadzu GC-MS, were designed to obtain more kinds of VOCs in *T. castaneum*. A comparison of the results revealed that some of the major compounds detected in the two sets of experiments were the same, with some differences.

**Figure 1:**
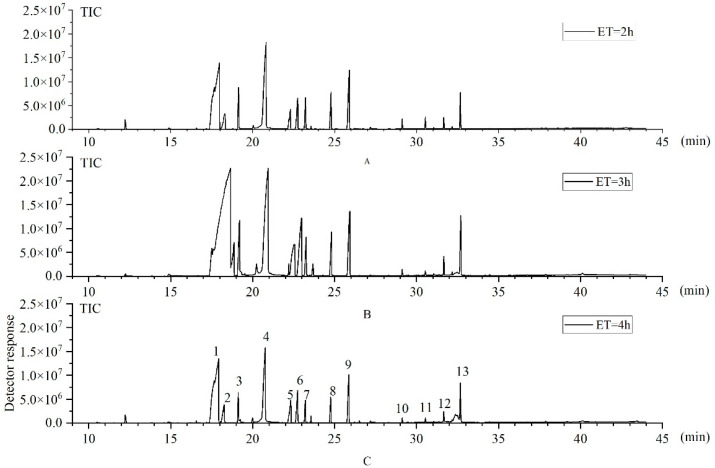
Representative chromatograms of VOCs based on DVB/CAR/PDMS + Agilent GC-MS + different extraction time conditions of *T. castaneum*. (**A**) 2 h, (**B**) 3 h, (**C**) 4 h. (1) 1-pentadecene, (2) 1-hexadecanol, (3) 2-methyl-p-benzoquinone, (4) 2-ethyl-p-benzoquinone, (5) 1-hexadecene, (6) cis-9-tetradecen-1-ol, (7) unknown, (8) unknown, (9) unknown, (10) m-cresol, (11) 3-ethylphenol, (12) paeonol, (13) 1-(2-hydroxy-4-methoxyphenyl)propen-1-one.

Figure 1 shows the representative chromatograms of three samples of *T. castaneum* with extraction times of 2 h, 3 h and 4 h under DVB/CAR/PDMS and Agilent GC-MS conditions, respectively. The results showed that the number and species of the main compounds obtained at different extraction times were the same. The relative content of the thirteen VOCs identified by GC-MS from *T. castaneum* was shown in Table 1.

### 3.2. Optimum Extraction Time

**Figure 2:**
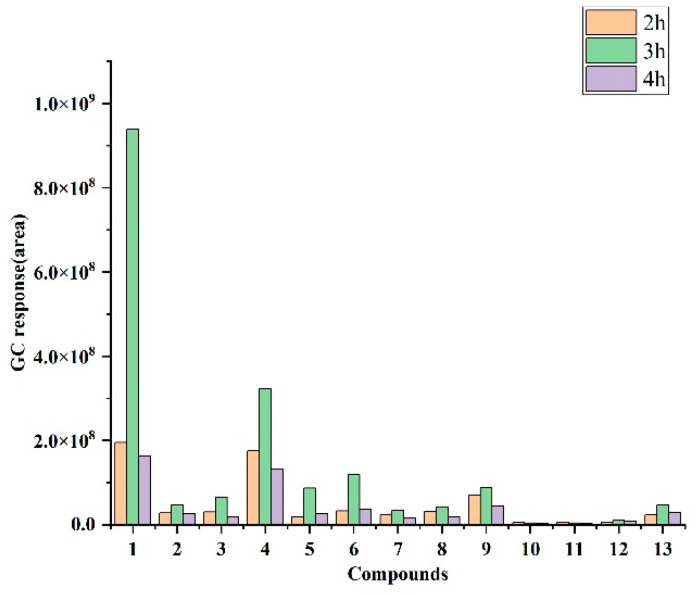
Peak areas of 10 VOCs based on DVB/CAR/PDMS + Agilent GC-MS + different extraction time conditions for *T. castaneum*.

Figure 2 presents the variations in peak areas for 13 main VOCs using the DVB/CAR/PDMS + Agilent GC-MS + various extraction time conditions for *T. castaneum*. The compounds were qualitatively analyzed: The compound species were the same for the 2 h, 3 h and 4 h extraction times. The content of VOCs was calculated by the semi-quantitative method: the extraction time of 3 h compared to 2 h showed a substantial increase in compound content; the extraction time of 4 h compared to 3 h showed a reduction in all compounds. The extraction time of 3 h was the highest for most compounds compared to 2 h and 4 h. When the extraction time was 3 h, the order of compound concentration from large to small was (1) 1-pentadecene, (4) 2-ethyl-p-benzoquinone, (6) cis-9-tetradecen-1-ol, (9) unknown, (5) 1-hexadecene, (3) 2-methyl-p-benzoquinone, (13) 1-(2-hydroxy-4-methoxyphenyl)propen-1-one, (2) 1-hexadecanol, (8) unknown, (7) unknown, (12) paeonol, (11) 3-ethylphenol, (10) m-cresol. At the extraction times of 2 h, 3 h and 4 h, the major compounds of *T. castaneum* had been obtained to satisfy the need for qualitative analysis of *T. castaneum*. The peak area of most of the VOCs increased with the increase in extraction time from 0 h, and the extraction equilibrium was nearly reached at 3 h. Therefore, the best extraction time for the semi-quantitative analysis of *T. castaneum* was 3 h.

### 3.3. Shimadzu GC-MS Analysis

**Figure 3:**
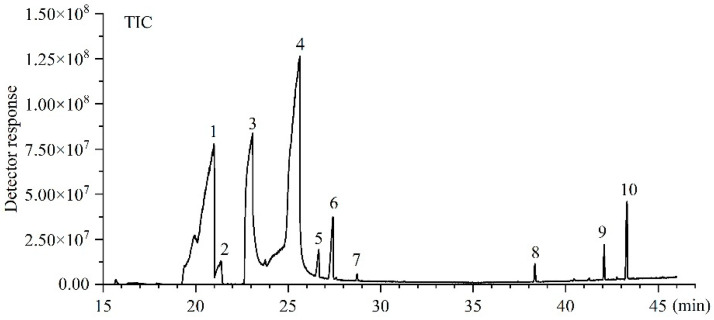
Representative chromatograms of VOCs based on CAR/PDMS+Shimadzu GC-MS + 3 h extraction time conditions of *T. castaneum*. (1) 1-pentadecene, (2) cyclododecene, (3) 2-methyl-p-benzoquinone, (4) 2-ethyl-p-benzoquinone, (5) 1-hexadecene, (6) cis-9-tetradecen-1-ol, (7) unknown, (8) m-cresol, (9) paeonol, (10) 4-ethoxy-3-anisaldehyde.

The *T. castaneum* sample represented 10 volatile chemicals according to the extraction time of 3 h, CAR/PDMS, and Shimadzu GC-MS (Figure 3).

### 3.4. Comparison of Analysis Results

In comparison with Table 2 and Table 3, a total of 16 major volatile compounds of *T. castaneum* were measured under polar column conditions, using two types of SPME fibers and two instruments, 12 of which were also detected by polar or non-polar columns by Niu, Alnajim et al. [25,36,37]. In this study, four compounds could not be identified. Seven common compounds were measured under different experimental conditions, which were 1-pentadecene, 2-methyl-p-benzoquinone, 2-ethyl-p-benzoquinone, 1-hexadecene, cis-9-tetradecen-1-ol, m-cresol and paeonol.

## 4. Discussion

The matrix in the sample can mask, absorb, or trap VOCs, thereby reducing their levels in the headspace. The study was carried out to detect *T. castaneum* only in order to reduce the impact of grain odor and the above-mentioned parameters on insect odor.

Several reports have suggested that differences in extraction times, SPME, manufacturers of GC-MS equipment, columns and other key test conditions can affect the detection of cereal worm compounds. To search for more polar and non-polar compounds to be found in *T. castaneum*, its secretions were examined in the study using different types of key experimental components and GC-MS instruments from different manufacturers. One of the aims of this study was to select a few specific compounds from VOCs of *T. castaneum* as the criteria to judge whether there were pests in the granary. The current study found that an appropriate increase in extraction time allowed higher concentrations of compounds to be obtained, which facilitated both qualitative and semi-quantitative analyses of *T. castaneum*. The evidence from this study confirmed that the optimal extraction time for determining the volatile organic compound composition of *T. castaneum* was 3 h using an HS-SPME/GC-MS technique.

In this study, a total of 16 major volatile compounds of *T. castaneum* were detected through two sets of experiments. Thirteen major volatile compounds were measured by an Agilent instrument using a three-phase solid-phase microextraction fiber membrane (DVB/CAR/PDMS). Ten major volatile compounds were measured by a Shimadzu instrument using a two-phase solid-phase microextraction fiber membrane (CAR/PDMS). Seven major VOCs were detected under both experimental conditions, which are 1-pentadecene, 2-methyl-p-benzoquinone, 2-ethyl-p-benzoquinone, 1-hexadecene, cis-9-tetradecen-1-ol, m-cresol and paeonol.

Consistent with this finding, several studies have also shown that 1-pentadecene, 2-methyl-p-benzoquinone, and 2-ethyl-p-benzoquinone are the main volatile organic compounds of Tribolium. These three compounds were also present at the highest levels in this study. cis-9-tetradecen-1-ol and paeonol have been detected [24,25].

Nikola Đukić concluded that 1-pentadecene was present only in wheat bran infested with *T. castaneum* and that low, medium and high concentrations of 1-pentadecene were attractive, neutral and repulsive to *T. castaneum*, respectively [29]. Niu [24,25] studied VOCs in wheat, flour, *R. dominica*, *T. castaneum*, wheat infested with *R. dominica*, and flour after infestation with *T. castaneum*. Twenty-three and thirty volatile compounds were identified by non-polar and polar chromatography columns in the *T. castaneum*, respectively. A total of 114 volatiles were identified in healthy wheat, *R. dominica*, and wheat infested with *R. dominica*. None of the 7 specific VOCs measured in this study was among the 114 volatiles of *T. castaneum*. It indicates that these seven compounds are not present in wheat and *R. dominica*. Villaverde measured 2-methyl-p-benzoquinone, 2-ethyl-p-benzoquinone and 1-pentadecene as the main volatile defense secretions of the *T. castaneum* [22]. Ihab Alnajim measured the main volatiles in *T. castaneum*, which are 1-pentadecene, 2-methyl-p-benzoquinone, and 2-ethyl-p-benzoquinone [36]. Tian XM concluded that 1-pentadecene could be a potential volatile biomarker for the presence of *T. castaneum* during the transport of brown rice [21]. Madhurya Lokesh detected 2-ethyl-p-benzoquinone in *T. castaneum* but not in *Callosobruchus maculatus* (Pulse beetle) and *Sitophilus oryzae* (Rice weevil) [38].

## 5. Conclusions

This study has shown that these seven compounds are not present in a variety of stored grains (flour, wheat and *R. dominica*, etc.) and grain pests; thus, these compounds can be used as VOCs specific to *T. castaneum* and can be used as indicators to determine whether stored grains are infected by *T. castaneum*. This research result could provide a reference for other grain storage pest detection technologies, for example, optimizing the E-nose detection method for grain storage pests, selecting sensors sensitive to seven compounds or making new sensors, improving the accuracy of individual sensors or sensor arrays, and ultimately improving the accuracy of E-nose detection. The process of this study could also provide a technical reference for the detection of flavor and mildew in food products to ensure food safety and the food safety of consumers.

## Figures and Tables

**Table 1:** Relative content of VOCs in *T. castaneum* based on DVB/CAR/PDMS + Agilent GC-MS + different extraction time conditions.

RT	VOC	RI	Samples (Extraction Time)			Rfs
			2 h	3 h	4 h	
17.4	1-pentadecene	1492	+++++++	+++++++	+++++++	*
18.0	1-hexadecanol	1880	+++	+++	+++	*
19.1	2-methyl-p-benzoquinone	1018	+++	++++	+++	*
20.7	2-ethyl-p-benzoquinone	-	++++++	++++++	++++++	*
22.1	1-hexadecene	1602	+++	++++	+++	*
22.6	cis-9-tetradecen-1-ol	1867	++	+++++	++++	*
23.2	-	-	+++	+++	+++	-
24.7	-	-	+++	+++	+++	-
25.7	-	-	+++++	++++	+++++	-
29.1	m-cresol	1075	+	+	+	*
30.5	3-ethylphenol	1169	+	+	+	*
31.7	paeonol	1438	+	+	++	*
32.6	1-(2-hydroxy-4-methoxyphenyl)propen-1-one	-	+++	+++	++++	*

RT, retention time (min); RI, retention indices; Rfs, * means the VOC also detected by Niu and Alnajim [25,36]. Trace level: tr, <1%; +, 1–5%; ++, 5–10%; +++, 10–20%; ++++, 20–30%; +++++, 30–50%; ++++++, 50–100%; +++++++, >100%; - means unknown. For *T. castaneum* with 2 h, 3 h, 4 h extraction time, the percentages were calculated using the total ion current (TIC) for each compound relative to the Tic 2-Ethyl-p-benzoquinone that recorded 2-Ethyl-p-benzoquinone = 100%.

**Table 2:** Information of 10 compounds based on DVB/CAR/PDMS + Agilent GC-MS + 3 h extraction time conditions.

Peak	RT	VOC	RI	CAS	Structure	Rfs
1	17.4	1-pentadecene	1492	13360-61-7	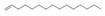	*
2	18.0	1-hexadecanol	1880	36653-82-4	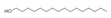	*
3	19.1	2-methyl-p-benzoquinone	1018	553-97-9	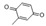	*
4	20.7	2-ethyl-p-benzoquinone	-	4754-26-1	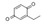	*
l5	22.1	1-hexadecene	1602	629-73-2	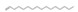	*
6	22.6	cis-9-tetradecen-1-ol	1867	35153-15-2	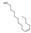	*
7	23.2	-	-	-	-	-
8	24.7	-	-	-	-	-
9	25.7	-	-	-	-	-
10	29.1	m-cresol	1075	108-39-4	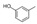	*
11	30.5	3-ethylphenol	1169	620-17-7	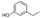	*
12	31.7	paeonol	1438	552-41-0	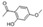	*
13	32.6	1-(2-hydroxy-4-methoxyphenyl)propen-1-one	-	6270-44-6	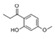	*

RT, retention time (min); RI, retention indices; CAS, CAS Number; Rfs, * means the VOC also detected by Niu and Alnajim [25,36]; - means unknown.

**Table 3:** Information of 9 compounds based on CAR/PDMS + Shimadzu GC-MS + 3 h extraction time conditions.

Peak	RT	VOC	RI	CAS	Structure	Rfs
1	19.9	1-pentadecene	1502	13360-61-7	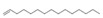	*
2	21.38	cyclododecene	1421	1501-82-2	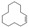	*
3	23.06	2-methyl-p-benzoquinone	1116	553-97-9	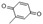	*
4	25.57	2-ethyl-p-benzoquinone		4754-26-1	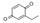	*
5	26.65	1-hexadecene	1602	629-73-2	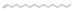	*
6	27.41	cis-9-tetradecen-1-ol	1667	35153-15-2	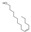	*
7	28.8	-	-	-	-	-
8	38.33	m-cresol	1075	108-39-4	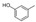	*
9	42.08	paeonol	1438	552-41-0	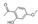	*
10	43.31	4-ethoxy-3-anisaldehyde	1460	120-25-2	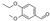	*

RT, retention time (min); RI, retention indices; CAS, CAS Number; Rfs, * means the VOC also detected by Niu, Alnajim and Huang J.W. [25,36,37]; - means unknown.

## Data Availability

The data in this study were available from the following sources the corresponding authors. These data are not publicly available due to the requirement to fund research research projects.
